# Bone Marrow-Derived Mononuclear Cell Transplants Decrease Retinal Gliosis in Two Animal Models of Inherited Photoreceptor Degeneration

**DOI:** 10.3390/ijms21197252

**Published:** 2020-09-30

**Authors:** Johnny Di Pierdomenico, Diego García-Ayuso, María Elena Rodríguez González-Herrero, David García-Bernal, Miguel Blanquer, José Manuel Bernal-Garro, Ana M. García-Hernández, Manuel Vidal-Sanz, María P. Villegas-Pérez

**Affiliations:** 1 Departamento de Oftalmología, Facultad de Medicina, Universidad de Murcia, 30120 Murcia, Spain; johnnydp@um.es (J.D.P.); jmbg@um.es (J.M.B.-G.); manuel.vidal@um.es (M.V.-S.); 2 Instituto Murciano de Investigación Biosanitaria-Virgen de la Arrixaca (IMIB-Arrixaca), 30120 Murcia, Spain; mariaelenargh@gmail.com (M.E.R.G.-H.); david.garcia23@um.es (D.G.-B.); miguelblanquer@gmail.com (M.B.); amgh8@hotmail.com (A.M.G.-H.); 3 Servicio de Oftalmología, Hospital Clínico Universitario Virgen de la Arrixaca, Carretera Madrid-Cartagena s/n, 30120 El Palmar Murcia, Spain; 4 Servicio de Hematología y Hemoterapia, Hospital Clínico Universitario Virgen de la Arrixaca, Carretera Madrid-Cartagena s/n, 30120 El Palmar Murcia, Spain

**Keywords:** inherited photoreceptor degeneration, intravitreal transplant, subretinal transplant

## Abstract

Inherited photoreceptor degenerations are not treatable diseases and a frequent cause of blindness in working ages. In this study we investigate the safety, integration and possible rescue effects of intravitreal and subretinal transplantation of adult human bone-marrow-derived mononuclear stem cells (hBM-MSCs) in two animal models of inherited photoreceptor degeneration, the P23H-1 and the Royal College of Surgeons (RCS) rat. Immunosuppression was started one day before the injection and continued through the study. The hBM-MSCs were injected in the left eyes and the animals were processed 7, 15, 30 or 60 days later. The retinas were cross-sectioned, and L- and S- cones, microglia, astrocytes and Müller cells were immunodetected. Transplantations had no local adverse effects and the CD45+ cells remained for up to 15 days forming clusters in the vitreous and/or a 2–3-cells-thick layer in the subretinal space after intravitreal or subretinal injections, respectively. We did not observe increased photoreceptor survival nor decreased microglial cell numbers in the injected left eyes. However, the injected eyes showed decreased GFAP immunoreactivity. We conclude that intravitreal or subretinal injection of hBM-MSCs in dystrophic P23H-1 and RCS rats causes a decrease in retinal gliosis but does not have photoreceptor neuroprotective effects, at least in the short term. However, this treatment may have a potential therapeutic effect that merits further investigation.

## 1. Introduction

Photoreceptor degenerative diseases can be inherited or acquired and be caused by genetic abnormalities or other less well-known nutritional or environmental factors [1,2]. These diseases are at present, the first cause of irreversible blindness in developed countries and one of the leading causes of irreversible blindness in the world [3,4,5,6].

By far, the most frequent inherited retinal degeneration is retinitis pigmentosa (RP), that has a prevalence of around 1 in 4000 individuals [7] and comprises a group of different diseases caused by mutations in more than 300 genes and loci [7,8,9]. However it is believed that there are more yet-unidentified mutations [7,8,9]. These mutations affect rod genes encoding proteins for a variety of cellular processes (i.e., metabolism or ciliary structure and function), including phagocytosis (i.e., MERTK, see below) and rod phototransduction [7,10,11]. The disease is usually limited to the retina, where it primarily causes rod loss and the patients experience nyctalopia but secondarily cone loss and blindness [7]. Why cones die following rods in RP is still a matter of consideration [12,13,14,15].

Age-related macular degeneration (AMD) is at present the first cause of irreversible blindness in adults in developed countries and its incidence is increasing due to the rise in life expectancy [16]. The disease causes the degeneration and death of the photoreceptors and retinal pigment epithelial (RPE) cells of the central retina (macula), and in some cases neovascular invasion of the retina causing central vision loss. AMD is a multifactorial disease that has both genetic (a large number of risk variants and loci [17]) and lifestyle (i.e., smoking, diet…) epidemiological factors [10,18]. This disease is currently an important health concern due to the growing numbers of affected persons and the costly therapy of the neovascular form, the less frequent but only treatable form of the disease [18].

Photoreceptor degenerations, independent of their cause, lead with time to secondary changes in the inner retina, such as rewiring, cell migration and neurodegeneration—a process known as retinal remodeling that may have adverse effects on retinal structure [15,19,20,21,22,23,24,25,26,27]. Thus, it is now recognized that it is important to halt the degeneration process before the inner retina is affected.

The optimal treatment for photoreceptor degeneration would be prevention of photoreceptor death. Since both in RP and AMD there are many genetic defects involved, gene therapy may not be as useful as it has been in other retinal diseases with few known genetic defects, such as Leber congenital amaurosis [10]. Thus, the research efforts have been directed to develop pharmacological or cellular therapies that could slow photoreceptor loss and/or replace the lost photoreceptors [9,10,28,29,30,31,32]. Stem cells (SC) can theoretically perform both these actions [33,34]. These cells can divide and differentiate into different cell types and, if transplanted to the retina, may substitute the damaged or lost retinal cells [35,36] and also secrete different factors that may prevent neuronal death [37]. SC can be obtained from different accessible tissues such as bone marrow, blood and adipose tissue [10,37,38,39]. Two types of cells, mesenchymal stem cells or mononuclear/CD34+ stem cells, can be harvested from the bone marrow aspirate and have shown promising results in different animal models of neuronal degeneration [40,41,42,43,44], including photoreceptor degeneration [39,43,45,46,47,48]. In this article, we use adult human bone-marrow-derived mononuclear/CD34+ stem cells (hBM-MSCs), a fraction that contains a small percentage of hematopoietic, mesenchymal and endothelial stem cells but also monocytes and lymphocytes, between other cells although more than 70% of these cells are CD34+ [43]. The hBM-MSCs have been used in clinical trials to induce angiogenesis in myocardial, peripheral or other types of ischemia [49,50]. Although these cells have not been documented to differentiate in other cell types, they release different factors and show beneficial immunomodulatory and regenerative properties [37,50]. Thus, hBM-MSCs have been used with apparent vascular regenerative capabilities in animal models of retinal ischemia [44,51,52].

Several studies performed in animal models of retinal degeneration have shown that hBM-MSCs transplantation to the eye can integrate in the retinas, rescue photoreceptors and improve visual function [45,48,53,54]. Based on these results, there have been a small number of clinical trials studying hBM-MSC transplantation to the eye to treat retinal degenerative diseases [9,43]. Some of them have shown modest visual improvements and lack of adverse effects [55,56,57,58,59]. Traditionally, studies investigating the effects of BM-MSCs therapies for retinal degenerations have used two means of cell delivery: (i) intravitreal injections, in which cells are delivered into the vitreous close to the internal retina, have shown promising retinal ganglion cell rescue effects [46,55,58] and (ii) subretinal injections, in which cells are delivered under the retina and thus close to the outer retina and photoreceptors, and have shown variable cell rescue outcomes [39,46,60]. In addition, the results with animal models are highly variable depending on several factors such as the delivery method, the age of the animals, the strain used or the method of analysis [45,48,53,54]. Therefore, further studies are needed to understand the survival, connectivity and functionality of grafted cells. Considering all of the above, our hypothesis is that intravitreal- or subretinal-injected hBM-MSCs could be a safe and effective cell therapy for retinal degenerations.

In this study we use intravitreal and subretinal transplantation of hBM-MSCs in two different animal models of inherited photoreceptor degeneration to investigate the safety, integration and possible rescue effects of these cells. Therefore, one main objective of this study is to ascertain whether intravitreal injections (IVI) or subretinal injections (SRI) of hBM-MSCs could be neuroprotective in two animal models of inherited photoreceptor degeneration with different etiologies. This study was planned as a safety study for a clinical trial: EudraCT 2012-000618-12.

## 2. Results

### 2.1. Safety and Feasibility of Intravitreal and Subretinal Injections

**Figure 1:**
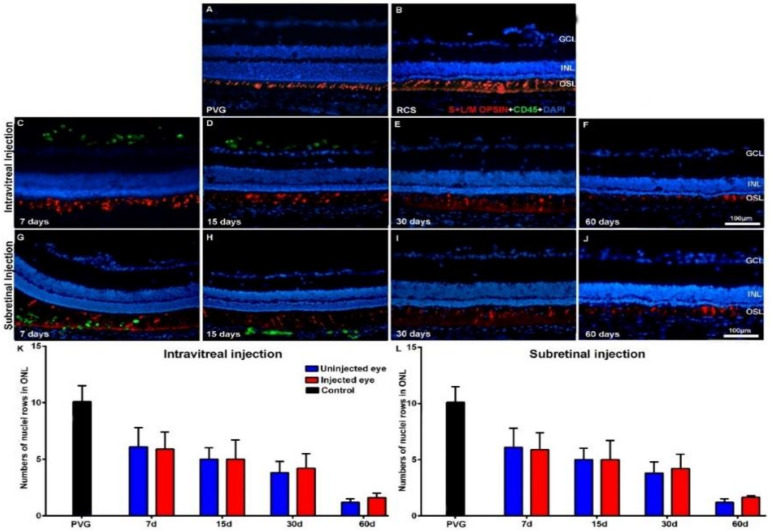
Cone morphology and ONL thickness in transplanted RCS rats. Microphotographs of representative retinal cross-sections taken from control Pievald Viro Glaxo (PVG) rats (**A**), and the right untreated eyes (**B**) and left treated eyes (**C**–**J**) of RCS rats that received IVI (**C**–**F**) or SRI (**G**–**J**) of hBM-MSCs. Immunostained cones (red; rabbit anti-red/green opsin and goat anti-blue opsin antibodies) and transplanted cells (green; mouse anti-human CD45 antibody) and also DAPI counterstaining (blue) of the retinas can be observed at different time periods after the injection. Graphs show the mean numbers ± SD of nuclei rows in the ONL of control PVG rats (black bars; include data from both right and left eyes) and in the right uninjected (blue bars) and left eyes (red bars) of RCS rats that received IVI (**K**) or SRI (**L**) of hBM-MSCs. No significant differences were observed between right and left eyes at any period. *n* = 6 eyes for control group and treated eyes group at all time points studied; *n* = 12 eyes for untreated eyes group at all time points studied. Scale bar: 100 μm. GCL = ganglion cells layer; INL = inner nuclear layer; OSL = photoreceptors outer segment layer; ONL = outer nuclear layer; hBM-MSCs = human bone-marrow-derived mononuclear/CD34+ stem cells; RCS = Royal College of Surgeons; IVI = intravitreal injections; SRI = subretinal injections.

**Figure 2:**
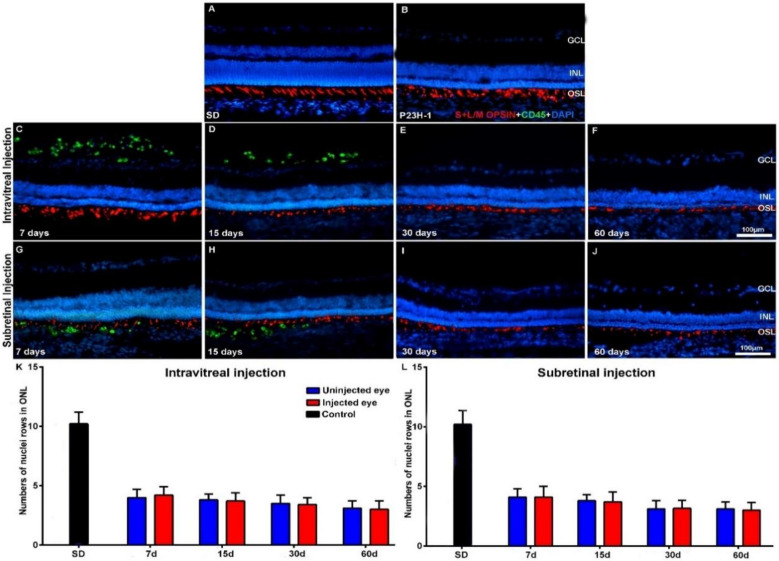
Cone morphology and ONL thickness in transplanted P23H-1 rats. Microphotographs of representative retinal cross-sections taken from control Sprague-Dawley (SD) rats (**A**), and the right untreated eyes (**B**) and left treated eyes (**C**–**J**) of P23H-1 rats that received IVI (**C**–**F**) or SRI (**G**–**J**) of hBM-MSCs. Immunostained cones (red; rabbit anti-red/green opsin and goat anti-blue opsin antibodies) and transplanted cells (green; mouse anti-human CD45 antibody) and also DAPI counterstaining (blue) of the retinas can be observed at different time periods after the injection. Graphs show the mean numbers ± SD of nuclei rows in the ONL of control SD rats (black bars; include data from both right and left eyes) and in the right uninjected eyes (blue bars) and left eyes (red bars) of P23H-1 rats that received IVI (**K**) or SRI (**L**) of hBM-MSCs. No significant differences were observed between right and left eyes at any period. *n* = 6 eyes for control group and treated eyes group at all time points studied; *n* = 12 eyes for untreated eyes group at all time points studied. Scale bar: 100 μm. GCL = ganglion cell layer; INL = inner nuclear layer; OSL = photoreceptors outer segment layer.

We did not find gross morphological abnormalities in the retinal cross-sections of the Royal College of Surgeons (RCS) (Figure 1) or P23H (Figure 2) rats that had received IVI or SRI of Phosphate-Buffered Saline (PBS) or hBM-MSCs. The presence of retinal detachment could not be ascertained because the retina normally appears somewhat detached in cryostat cross-sections. We did not observe microscopic abnormalities—presence of inflammatory cells, tumors or epiretinal membrane formation in the retinas of the experimental animals.

In the animals that had received IVI of PBS, we found a normal interface between the vitreous and the retina. In the animals that received IVI of hBM-MSCs, we observed clusters of CD45^+^ cells in the vitreous, most of them attached to the inner retinal limiting membrane (Figure 1C,D and Figure 2C,D). These cells were seen in both animal strains (RCS and P23H-1) for up to 15 days after injection (Figure 1C,D for RCS and Figure 2C,D for P23H-1). In the animals that received SRI of hBM-MSCs, CD45^+^ cells were found forming a 2–3-cells-thick layer in the subretinal space also during the first 15 days after the injection (Figure 1G,H for RCS and Figure 2G,H for P23H-1). We did not find hBM-MSCs-CD45^+^ in the vitreous or in the subretinal space of the animals analyzed 30 or 60 days after the IVI (Figure 1E,F for RCS and 2E,F for P23H-1) or SRI (Figure 1I,J for RCS and Figure 2I,J for P23H-1). We did not observe CD45+ integrated in the retina of the experimental animals at any survival period.

### 2.2. Effect of Intravitreal and Subretinal Injections in Photoreceptor Degeneration and Retinal Gliosis

The mean numbers (± standard deviation) of nuclei rows in the ONL in control Pievald Viro Glaxo (PVG) and Sprague-Dawley (SD) rats were 10.4 ± 1.2 (Appendix A, Table A1) and 10.2 ± 1.1 (Table A1), respectively, at 21 days of age, and 10.1 ± 1 (Table A1) and 10.5 ± 1.4 (Table A1), respectively, at 81 days of age. These ages were chosen because they correspond to the ages of the animals in the experimental groups 7 and 60 days after the injection. No significant differences in the numbers of ONL nuclei rows were found between the two control strains at these ages. No significant differences were found in the numbers of ONL nuclei rows between these survival intervals within the same strain. Thus, the numbers of nuclei rows of the ONL were similar in both control strains at the ages analyzed and did not decrease with age.

The mean numbers of nuclei rows of the ONL in the right uninjected eyes of RCS and P23H-1 rats that received IVI in the other eye were 6.1 ± 1.7 and 4 ± 1 at 7 days after the injection—these numbers were significantly lower than those found in the eyes of control animals (Table A1). The mean numbers of ONL nuclei were at this time significatively higher in the right uninjected eyes in RCS rats than in P23H-1 rats (Table A1). This significant difference between the RCS and the P23H-1 rats in the numbers of rows of the ONL was maintained throughout the study up until the last age analyzed (Table A1), and thus, from 21 and 81 days of age, photoreceptor cell-loss proceeds more slowly in RCS rats than in P23H-1 rats. Specifically, the number of nuclei rows of the ONL in the right uninjected eyes of the animals with retinal degeneration that received IVI in the left eyes decreased significantly during the period of study: from 6.1 ± 1.7 to 1.2 ± 0.3 in RCS rats (Table A1; Figure 1K,L) and from 4 ± 1 to 3.1 ± 0.8 in P23H rats (Table A1; Figure 2K,L) between the youngest and the oldest age analyzed, respectively. This significant decrease in the numbers of nuclei rows of the ONL was observed in the right and the left eyes of all the experimental groups between 7 and 60 days after the injection, indicating progressive photoreceptor death with age in both eyes of all the experimental animals.

One of the main objectives of this study was to ascertain whether the IVI or the SRI of hBM-MSCs could be neuroprotective and prevent photoreceptor degeneration in the injected eyes. For this purpose, we compared the numbers of nuclei rows of the ONL between the right (contralateral) eyes and the left (injected) eyes in the different groups of animals at the same survival intervals and we did not observe any significant differences, independent of the substance injected (PBS or hBM-MSCs) or the injection type (IVI or SRI), nor in RCS (Table A1; Figure 1K,L) or P23H (Table A1; Figure 2K,L) rats. Thus, we were not able to show increased photoreceptor survival in the left injected eyes during the period of study in both strains.

Finally, within the same strain (RCS or P23H-1) we compared the numbers of nuclei rows of the ONL between the left (PBS or hBM-MSCs) injected eyes of the animals that had received IVI or SRI and we did not find significant differences between them (Table A1). Thus, within the same strain, we failed to document differences in photoreceptor degeneration between the PBS or hBM-MSCs left injected eyes, for the IVI or the SRI and thus, we failed to document increased survival in the hBM-MSCs-injected eyes when compared with the PBS-injected eyes.

### 2.3. Effect of Intravitreal and Subretinal Injections in the Retinal Microglial Cells

**Figure 3:**
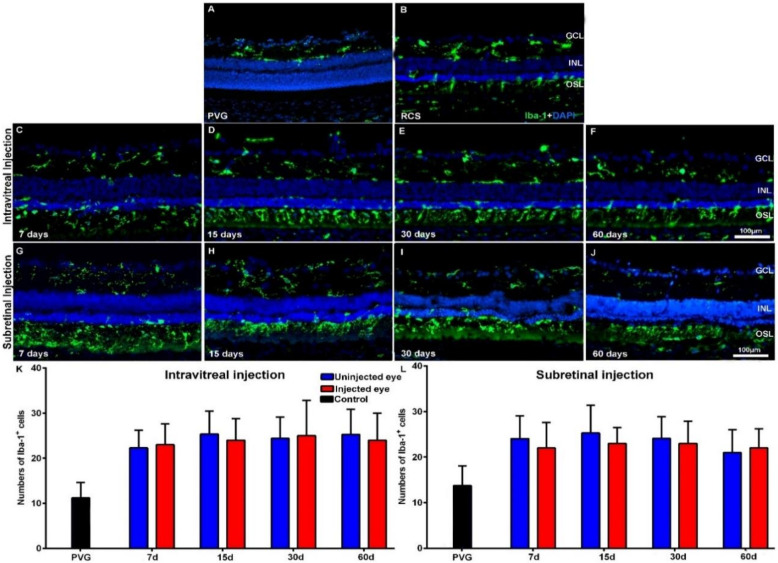
Microglial cell activation in transplanted RCS rats. Microphotographs of representative retinal cross-sections taken from control Pievald Viro Glaxo (PVG) rats (**A**), and the right untreated eyes (**B**) and left treated eyes (**C**–**J**) of RCS rats that received IVI (**C**–**F**) or SRI (**G**–**J**) of hBM-MSCs. Immunostained microglial cells (green; rabbit anti-Iba1 antibody) and also DAPI counterstaining (blue) of the retinas can be observed at different time periods after the injection. Graphs show the mean numbers ± SD of Iba-1^+^ cells in the retinas of control PVG rats (black bars; include data from both right and left eyes) and in the right uninjected eyes (blue bars) and left eyes (red bars) of RCS rats that received IVI (**K**) or SRI (**L**) of hBM-MSCs. No significant differences were observed between right and left eyes at any period. *n* = 6 eyes for control group and treated eyes group at all time points studied; *n* = 12 eyes for untreated eyes group at all time points studied. Scale bar: 100 μm. GCL = ganglion cells layer; INL = inner nuclear layer; OSL = photoreceptors outer segment layer.

**Figure 4:**
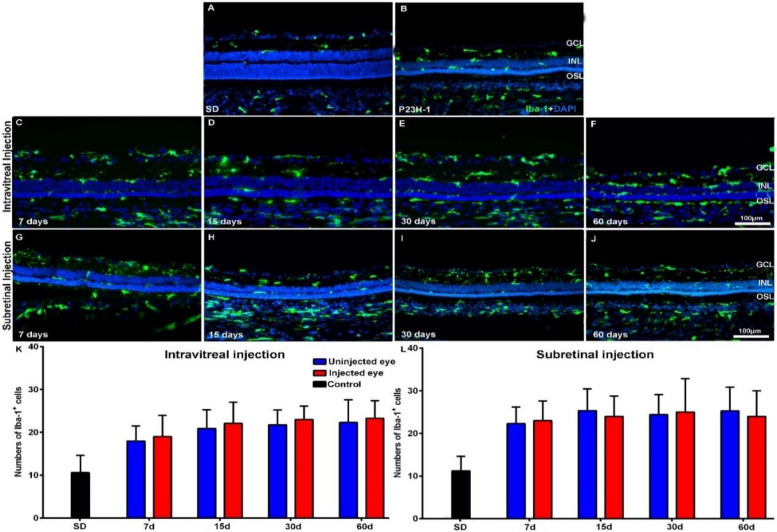
Microglial cell activation in transplanted P23H-1 rats. Microphotographs of representative retinal cross-sections taken from control Sprague-Dawley (SD) rats (**A**), and the right untreated eyes (**B**) and left treated eyes (**C**–**J**) of P23H-1 rats that received IVI (**C**–**F**) or SRI (**G**–**J**) of hBM-MSCs. Immunostained microglial cells (green; rabbit anti-Iba1 antibody) and also DAPI counterstaining (blue) of the retinas can be observed at different time periods after the injection. Graphs show the mean numbers ± SD of Iba-1+cells in the retina of control SD rats (black bars; include data from both right and left eyes) and in the right uninjected eyes (blue bars) and left eyes (red bars) of P23H-1 rats that received IVI (**K**) or SRI (**L**) of hBM-MSCs. No significant differences were observed between right and left eyes at any period. n=6 eyes for control group and treated eyes group at all time points studied; *n*=12 eyes for untreated eyes group at all time points studied. Scale bar: 100 µm. GCL= ganglion cells layer; INL= inner nuclear layer; OSL= photoreceptors outer segment layer.

The Iba-1^+^ cells of the retinas of the control animals showed a quiescent morphology and were located in both the plexiform layers, the retinal ganglion cell (RGC) layer and retinal nerve fiber RNF layer, as observed in normal animals [61]. The mean numbers of microglial cells in control animals increased from 11.3 ± 6.2 to 13.7 ± 4.7 in PVG rats and from 10.6 ± 4 to 14 ± 4.7 in SD rats during the period of study, but this increase was not statistically significant (Table A1). However, in the retinas of both RCS (Figure 3A,B) and P23H-1 (Figure 4A,B) rats, we found qualitative morphological signs of microglial activation and migration to the outer retinal layers.

The numbers of Iba-1^+^ cells in the right uninjected eyes of the animals with retinal degeneration were 7 days after injection 22.5 ± 3.9 or 24.1 ± 5 in RCS rats and 18 ± 3.5 or 22.3 ± 3.9 in P23H-1 rats—these numbers were significantly higher than those found in control retinas at the same age. The numbers of Iba-1^+^ cells in the right uninjected eyes of the experimental animals varied slightly during the period of study, but the variation was not statistically significant (Table A1). The numbers of Iba-1^+^ cells in the left injected eyes also varied but not significatively during the period of study. We did not observe qualitative changes in the morphology or location of the microglial cells between the left injected eyes and the right uninjected eyes: the IVI or SRI injections of PBS or of hBM-MSCs did not produce changes in the activation state (Figure 3 for RCS and Figure 4 for P23H-1) or the migration to the outer retinal layers.

When we compared the numbers of Iba-1^+^ cells between the right uninjected and left injected eyes (PBS or hBM-MSCs) of the experimental animals, we did not find significant differences between them, at any age, independently of the technique of injection (IVI or SRI) both in RCS (Table A1; Figure 3 K,L) and P23H-1 (Table A1; Figure 4K,L) rats.

We did not find significant differences in the numbers of Iba-1^+^ cells between the left (PBS or hBM-MSCs) injected eyes of the animals that had received IVI or SRI (Table A1). Thus, we failed to document differences in microglial cell activation between the PBS or hBM-MSCs left injected eyes, either for the IVI or the SRI, possibly due to the effect of immunosuppression on these cells [62,63,64].

### 2.4. Effect of Intravitreal and Subretinal Injections in the Retinal Macroglial Cells

**Figure 5:**
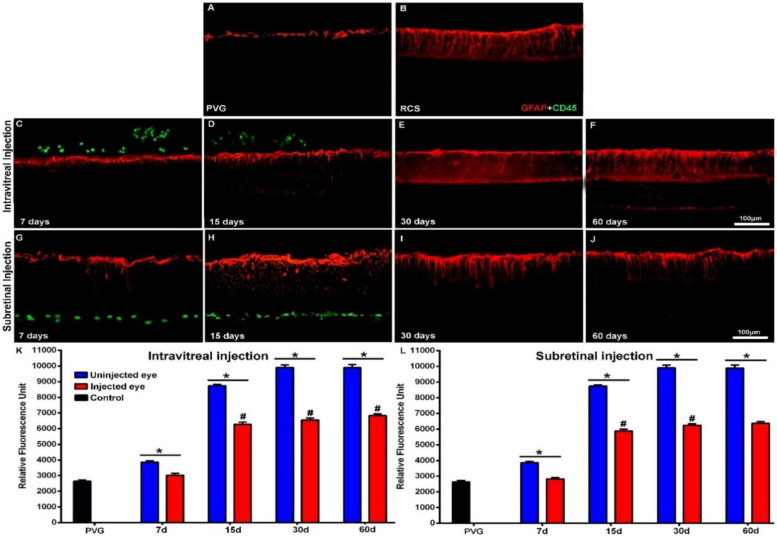
GFAP immunoreactivity in transplanted RCS rats. Microphotographs of representative retinal cross-sections taken from the control Pievald Viro Glaxo (PVG) rats (**A**), and the right untreated eyes (**B**) and left treated eyes (**C**–**J**) of RCS rats that received IVI (**C**–**F**) or SRI (**G**–**J**) of hBM-MSCs. Immunostaining for GFAP (red; goat anti-GFAP antibody), CD45 (transplanted cells; green; mouse anti-human CD45 antibody) and DAPI counterstaining (blue) of the retinas can be observed at different time periods after the injection. Graphs show the mean relative fluorescence units ± SD of GFAP immunofluorescence in the retinas of control PVG rats (black bars; include data from both right and left eyes) and in the right uninjected eyes (blue bars) and left eyes (red bars) of RCS rats that received an IVI (**K**) or SRI (**L**) of hBM-MSCs. GFAP immunoreactivity was significantly higher in the right (uninjected) eyes at all the survival periods. * *p* < 0.005, ^#^ *p* < 0.005 compared to previous survival interval. *n* = 6 eyes for control group and treated eyes group at all time points studied; *n* = 12 eyes for untreated eyes group at all time points studied. Scale bar: 100 μm.

**Figure 6:**
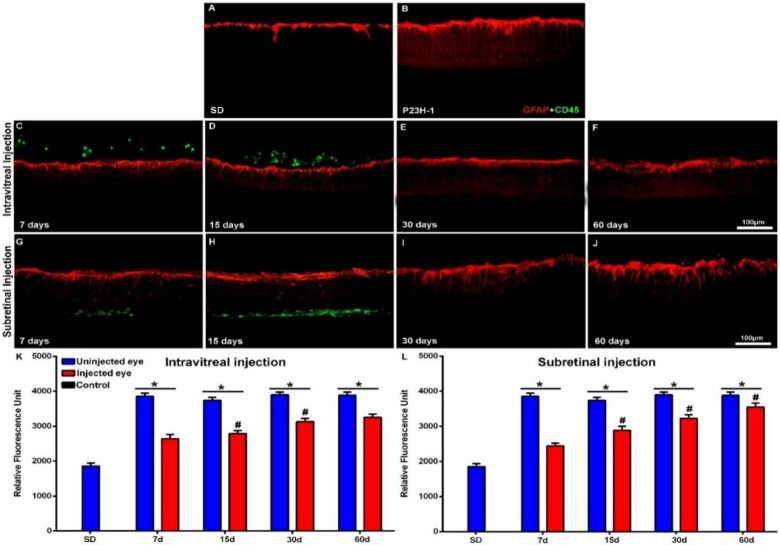
GFAP immunoreactivity in transplanted P23H-1 rats. Microphotographs of representative retinal cross-sections taken from the control Sprague-Dawley (SD) rats (**A**), and the right untreated eyes (**B**) and left treated eyes (**C**–**J**) of P23H-1 rats that received IVI (**C**–**F**) or SRI (**G**–**J**) of hBM-MSCs. Immunostaining for GFAP (red; goat anti-GFAP antibody), and CD45 (transplanted cells; green; mouse anti-human CD45 antibody) and DAPI counterstaining (blue) can be observed at different time periods after the injection. Graphs show the mean relative fluorescence units ± SD of GFAP immunofluorescence in the retinas of control SD rats (black bars; include data from both right and left eyes) and in the right uninjected eyes (blue bars) and left eyes (red bars) of P23H-1 rats that received an IVI (**K**) or SRI (**L**) of hBM-MSCs. GFAP immunoreactivity was significantly higher in the right (uninjected) eyes at all the survival periods. * *p* < 0.005, ^#^ *p* < 0.005 compared to previous time points. *n* = 6 eyes for control group and treated eyes group at all time points studied; *n* = 12 eyes for untreated eyes group at all time points studied. Scale bar: 100 μm.

In control retinas, GFAP immunoreactivity was limited to the astrocytes of the inner retina (Figure 5A and Figure 6A). In the animals with retinal degeneration, the cell bodies of Müller cells became strongly GFAP immunoreactive from the innermost retinal layer to the ONL (Figure 5B for RCS and Figure 6B for P23H-1), but this immunoreactivity varied between the strains and also depended on the substances injected.

To quantify GFAP expression, we calculated the Relative Fluorescence Unit (RFU) of the photographs. The RFU in control eyes was 1567 ± 8.7 and 1736 ± 96 in PVG and SD rats at 21 days of age and increased to 1987 ± 85 and 1856 ± 85 at 81 days of age, respectively. This increase was however, not significant (Table A1).

The right uninjected eyes of the experimental animals showed from 21 days of age higher RFU than control eyes. RFU at this age was 3856 ± 90 and 3987 ± 99 in RCS and P23H-1 rats, values that were significatively higher than those found in control eyes. However, at this age there were not significant differences in RFU between RCS and P23H-1 rats (Table A1). The RFU increased significatively with age in the right uninjected retinas of RCS rats but not in P23H-1 rats. This increase in immunoreactivity with age in RCS rats may be due to the fluorescence of photoreceptor debris accumulation.

The increase in RFU (GFAP) immunoreactivity in RCS rats occurred both in the right and the left eyes and occurred mainly between 7 and 14 days after injection (Table A1) and thus, from 14 days of age, RFU values were significantly higher in RCS rats than in P23H-1 at all the survival intervals.

The RFU values of the left injected eyes varied depending on the strain and the substance injected. We found in the left eyes that received IVI or SRI of PBS RFU values similar to those of the right uninjected eyes in both strains at all ages. However, we found in the left eyes that received IVI or SRI of hBM-MSCs significatively lower RFU values than those found in the right eyes. This decreased GFAP immunoreactivity in hBM-MSCs-injected eyes was due to decreased GFAP immunoreactivity in both astrocytes and Müller cells and was observed both in RCS (Figure 5) and P23H-1 (Figure 6) rats. The effect was similar with both injection techniques (IVI; Figure 5C–F for RCS and Figure 6C–F for P23H-1; and SRI; Figure 5G–J for RCS and Figure 6G–J for P23H-1). However, these decreases in GFAP immunoreactivity found in the hBM-MSCs-injected eyes were not sufficient to prevent the significant increases in RFU observed with age in both strains.

## 3. Discussion

In this work, we investigated the possible effects of intravitreal or subretinal injection of hBM-MSCs in the retinas of two dystrophic animals: pigmented RCS rats and albino P23H-1 rats. These are well-known models of retinal degeneration and both have genetic defects also observed in human disease [23,65]. Transplantation of hBM-MSCs to the RCS rat eye has been carried out before by other authors [45,47,60], but to the best of our knowledge, this is the first time that is investigated in the P23H-1 rat.

For comparison and in order to assess the cellular events observed in the strains with retinal degeneration, we used two strains of control animals: the pigmented PVG and the albino SD rats. We document, by comparison with the control animals, that the right uninjected eyes of the experimental animals show from age 28 (that is the earliest day of the study), a significant lower number of nuclei in the ONL, a significantly higher number of microglial cells and significantly higher GFAP immunofluorescence. Thus, at 28 days of age, photoreceptor death has commenced in both strains and is accompanied by microglial cell activation and proliferation and macroglial cell hypertrophy as reported by our group and others [24,66]. This is the reason why we chose this age to initiate this study, because at this age in P23H-1 rats there is loss of approximately 50% of the photoreceptors and in RCS loss has just started [15,24].

We performed the intraocular injections in animals with photoreceptor degeneration at 21 days of age just after weaning and observed the retinas between 28 and 81 days of age. In control animals from 28 to 81 days of age we did not observe significant variations of the number of nuclei rows in the ONL, the number of microglial cells in the retina or the RFU of the GFAP-immunostained retinas. Nevertheless, in the right uninjected eyes of the dystrophic animals we observed between 28 and 81 days of age significant decreases of the numbers of the ONL nuclei rows, but not significant increases of the number of microglial cells. Thus, although photoreceptor degeneration progressed in dystrophic animals during the period of study, the numbers of microglial cells remained stable. In a previous study we documented increased microglial cell numbers between P10 and P60 in the retinas of P23H-1 and RCS rats when compared with their control animals [24]. However, during the period of study, the increased numbers remained stable in P23H-1 but increased with age in RCS rats [24]. In the present study we quantify microglial cells in older animals, from P20 to P81, and show increased numbers of microglial cells in both dystrophic strains when compared with control animals. However, we did not find an increase in the numbers of microglial cells in the right uninjected eyes during the period of study in any of the dystrophic strains, and we wonder if this could be due to the systemic immunosuppression (see below). We also observed during the period of study in the GFAP-immunoreacted retinas a significant increase in RFU but only in one strain—the RCS rat, and this occurred mainly between 21 and 35 days. This increase may be due to increases in GFAP immunoreactivity of Müller cells and astrocytes but may also be due to increased fluorescence in the photoreceptor debris area [24].

It has been proven that, during chronic diseases, Astrocyte hypertrophy and Müller cell upregulation of cytoskeletal components such as GFAP occurs in response to inflammation in the retina, and that inhibition of reactive gliosis prevents apoptotic death of retinal neurons and provides substantial neuroprotection [67]. hBM-MSCs reduce gliosis by modulating tissue levels of pro-inflammatory cytokines through the release of factors with paracrine anti-inflammatory effects.

In the present study, we initiated immunosuppression at 20 days in all animals—control and dystrophic—one day prior to the intraocular injection and we continued it for the period of study. We used two different methods of injection in dystrophic animals—IVI and SRI—and document the safety of both delivery methods because we did not observe adverse effects. We also document that the transplanted cells persist in the eye next to the inner limiting membrane (ILM) or under the retina for at least 15 days after IVI or SRI, respectively, and this is in accordance with previously published work using similar delivery methods [46]. However, we did not find migration of the injected cells and integration within the retinal layers, in accordance with previous studies [65,68]. Since these studies did not perform immunosuppression [65,68], we wonder whether the limited survival and the lack of integration of the transplanted cells could have been due to cell rejection [43]. Nonetheless, for the neuroprotective effects of hBM-MSCs, transplantation may depend more on their paracrine and immunomodulatory properties [37,54,60] than in their integration within the retina.

One of the limitations of this study is therefore the injection of human cells in rat strains. Previous works have proposed that immunosuppression is not a prerequisite for achieving therapeutic benefit from hBM-MSCs transplantation into the rat retina [39]. Nevertheless, the question arises whether immunosuppression could improve survival times of hBM-MSCs xenograft [46] and differentiation into photoreceptors. Here we immunosuppressed transplanted rats with cyclosporine A and dexamethasone, but we did not observe long-term survival of the transplanted cells. This leads us to think that perhaps, the best option would be to perform autologous transplants or at least transplants from the same strain, since this could result in longer transplant survival and prolonged therapeutic effect [69,70,71]. In this work, transplanted cells could be identified only up to 15 days after the injection and the antigliotic effect lasted longer but lost effectiveness over time, which is in accordance with what has been published by other authors using similar delivery methods [46]. Another limitation of this study could be that we did not use molecular techniques to quantify GFAP expression. However, previous works have shown that there is a relationship between GFAP immunohistochemistry and GFAP expression in other animal models of retinal degeneration [72,73].

Some previous works have shown that treatments or insults in one eye may affect the contralateral eye [73,74,75,76,77], however others have failed to document contralateral effects [78,79]. To examine the possible effects of hBM-MSCs injection in the contralateral eye, we compared the data from the contralateral eyes with that of the eyes of the animals that received PBS, and we found no differences between them, which allows us to rule out a possible effect of the treatment on the contralateral eyes. Therefore, we assessed the possible neuroprotective effects of the injections on photoreceptor degeneration by counting the numbers of nuclei rows in the ONL in the left injected eyes and comparing them to the numbers found in the right uninjected eyes. Our results show that neither PBS nor hBM-MSCs injections increased the numbers of nuclei rows in the left injected eyes. Thus, we have not been able to show a significant rescue effect of the hBM-MSCs injections on photoreceptor survival, which is in disagreement with the findings of other studies using hBM-MSCs [45,46,47,60]. However, our results are in accordance with previously published studies that document that the IVI of hBM-MSC in humans has no adverse effects [55,58] and causes modest visual improvements [57,58].

Although in this study we did not find in dystrophic animals increases in microglial cell densities during the period of study, we did not find decreases in the numbers of microglial cells in the left injected eyes when we compared them with the right uninjected eyes. Thus, we failed to show an effect of the intraocular injections on microglial cell activation. However, we found that the left eyes that received IVI or SRI of hBM-MSCs had significantly lower RFU values and therefore decreased GFAP immunoreactivity in the retina and this was true in both strains—RCS and P23H-1 rats. This antigliotic effect was similar in both strains and with both injection techniques. Retinal gliosis is an early sign of photoreceptor degeneration [24,80] that may accompany intravitreal injections of substances [61] or of bone marrow mesenchymal stem cell [81] and includes microglial cell activation and migration to the outer retinal layers and hypertrophy and overexpression of GFAP in Müller cells and may have negative effects in retinal remodeling. Retinal degeneration proceeds with formation of a glial seal that can prevent the integration of the transplanted cells [27,80]. Furthermore, retinal gliosis may affect the structure of the entire retina causing neuronal death and impeding the success of treatments designed to replace or substitute photoreceptors [23,27,81] and therefore is important to prevent it.

The decreased retinal gliosis that we show after IVI or SRI of hBM-MSCs has been documented in previous studies using subretinal injections of hBM-MSCs [60] and hBM-derived somatic cells [39] in RCS rats. Although in this study we fail to document that the transplanted cells increase photoreceptor survival, we think that the intraocular transplantation of hBM-MSC shows promising effects highlighted in our study by an antigliotic effect, that was similar with both types of injections. Thus, we think that the effects of intraocular hBM-MSCs transplants should be further investigated because it may impede retinal remodeling [23] and therefore may be beneficial for patients with retinal degenerations, alone or in combination with other therapies, since it may also enhance the beneficial effects of the other treatments [60] and/or facilitate the outcomes of treatments aimed to replace photoreceptors. Finally, further studies are needed to provide more quantitative data (i.e., RT-PCR or Western blot analysis) about the described changes in GFAP expression that could clarify why it is not enough to protect photoreceptors.

## 4. Material and Methods

### 4.1. Animals

As experimental animals, we used female rats of two different strains with inherited retinal degeneration: the homozygous albino P23H line 1 (P23H-1) rats, obtained from M. LaVail (UCSF), and the homozygous pigmented female Royal College of Surgeons (RCS) rats from our own colony. These animals received intraocular injections in the left eyes (see below) and were processed 7, 15, 30 or 60 days later. Additional groups of female Sprague-Dawley (SD; as background for the P23H albino strain [64]) and Pievald Viro Glaxo (PVG; commonly used as control for the pigmented RCS strain [24,82]) rats were included as control. These groups are referred to as control groups all throughout the text and were untreated and age-matched to the experimental groups with the shortest and longest survival time.

All the animals were bred at the University of Murcia and housed in temperature- and light-controlled rooms with a 12-h light/dark cycle (light from 8 am to 8 pm) and had food and water ad libitum. The project was approved by the “Ethics Committee for Animal Testing” of our institution, the University of Murcia (ES300305440012; 254/2016; 04/28/2016). The human cells were obtained from healthy volunteers that donated bone marrow to the “Cell Bank of the Region of Murcia” (Biobanc-Mur) and authorized their use for this research project. Human material was obtained and treated following the principles of the Declaration of Helsinki. Animal manipulations were carried out following the Spanish and European Union regulations for the use of animals in research (Council Directive 86/609/EEC) and the Association for Research in Vision and Ophthalmology (ARVO) statement for the use of animals in ophthalmic and vision research.

All the control and experimental animals received immunosuppression that was achieved by the administration of cyclosporine A in drinking water (210 mg/L) from 1 day before transplantation until processing and also daily intraperitoneal injections of dexamethasone (1.6 mg/kg/day) from 1 day before transplantation and during 14 days, following previously described methods [83].

### 4.2. Human Bone-Marrow-Derived Mononuclear Cell Isolation

The bone marrow samples used for this study were obtained by percutaneous direct aspiration of bone marrow cells from the iliac crest of 5 healthy volunteers who were informed about the research and signed an informed consent. Bone marrow aspirate was placed in a sterile tube containing sodium heparin (20 U/mL) and was subjected to a Ficoll density gradient-based separation protocol to isolate the mononuclear cell fraction. The numbers of cells were counted in a Neubauer chamber and the cells were resuspended in sterile Phosphate-Buffered Saline to obtain a density of 50,000 cells/µL.

### 4.3. Cell Transplantation

**Figure 7:**
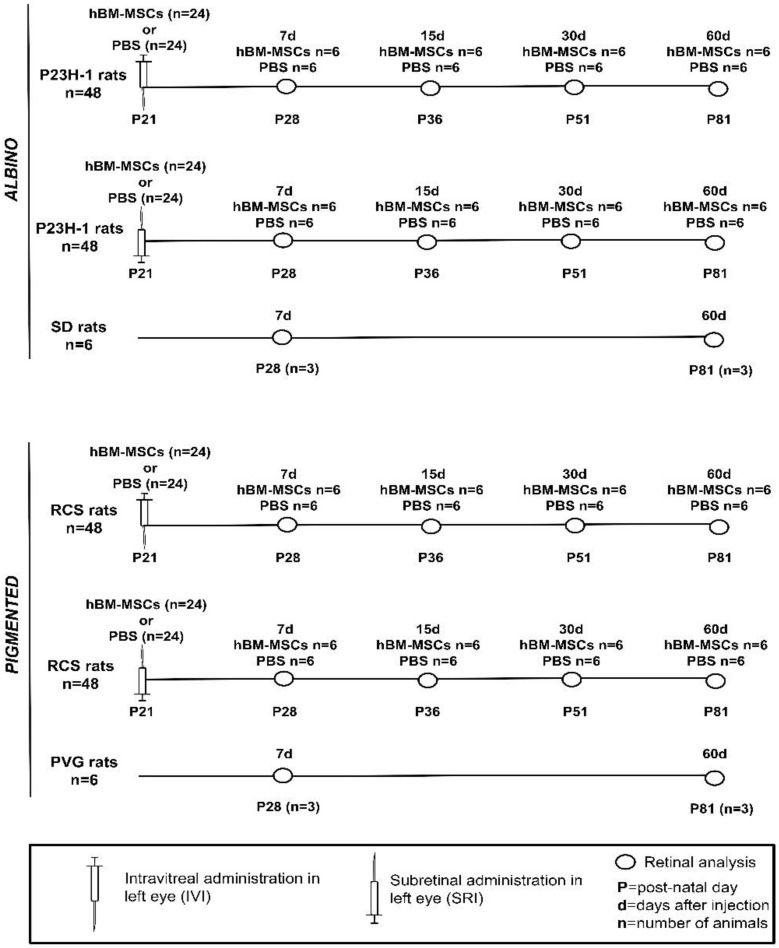
Experimental design. P23H-1 = P23H line 1. SD = Sprague-Dawley. RCS = Royal College of Surgeons. PVG = Pievald Viro Glaxo.

The experimental animals were divided into groups of 6 animals for each strain, type of injection, substance injected and time point studied (Figure 7). These animals received in the left eye, at post-natal day (P) 21, 5 µL intravitreal injections (IVI) or subretinal injections (SRI) of 0.25 × 10^6^ cells or of PBS. Animals were sacrificed 7, 15, 30 or 60 days after the injection. The animals that showed retinal injuries or developed cataracts during or after the injection were discarded.

IVIs were performed following previously described methods [84,85]. Briefly, a perforating sclerotomy was made in the superotemporal sclera with a sterile 27G needle at approximately 1–2 mm from the limbus. Then, the needle of a 26G Hamilton microsyringe was introduced towards the center of the globe with direct visualization through the operating microscope. When the tip of the needle was visualized in the vitreous, the injection was made and the needle retracted.

Transcleral SRI were performed through a perforating posteriorly oriented sclerotomy made just posterior to the limbus made using a 30-gauge needle and under direct visualization with the operating microscope. The tip of a 33-gauge blunt needle inserted on a Hamilton syringe (Hamilton, Reno, Nevada) was passed through the scleral hole directed posteriorly and the cells were slowly injected [86,87]. In previous experiments, trypan blue staining was used to verify the viability and integrity of the cells after passing through the 33G needle of the Hamilton syringe. Post-injection direct ophthalmoscopic examination was done through the operating microscope and most times in the early postoperative period a retinal detachment was visualized in the injected quadrant. The eye fundus was also inspected just before processing and no morphological abnormalities were found.

### 4.4. Tissue Processing

Rats were sacrificed with a lethal dose of sodium pentobarbital (Dolethal Vetoquinol, S.A., Lure, France) and perfused transcardially through the aorta first with saline and then with 4% paraformaldehyde in 0.1 M phosphate buffer (pH 7.4). Both eyes were enucleated and the eyecups included in Tissue-TEK, frozen and sectioned in the cryostat to obtain 15-microns-thick sagittal sections [24,74,87].

#### Immunohistofluorescence

Three cross-sections spanning the optic disk were selected per eye (right contralateral uninjected and left injected) and animal and processed for immunohistofluorescence using different antibody combinations and standard procedures [24,74,87]. Briefly, cross-sections were washed in PBS containing 0.1% Triton X-100 (Tx; Sigma-Aldrich, Alcobendas Madrid, Spain) and incubated overnight at 4°C with a mixture of the primary antibodies (see next paragraph) diluted in blocking buffer (PBS containing 2% Tx and 2% normal donkey or goat serum, depending on the origin of the secondary antibody used). After washing with PBS-0.1% Tx the sections were incubated for 1 h at room temperature with a mixture of the secondary antibodies (see next paragraph) diluted in PBS containing 2% Tx. Sections were rinsed again with PBS containing 0.1% Tx and then mounted with a mounting media containing DAPI (4′,6-diamidino-2-phenylindole; Vectashield Mounting Medium con DAPI, Vector Atom, Alicante, España) to counterstain all retinal nuclei.

### 4.5. Antibodies

Primary antibodies: hBM-MSCs were detected using FITC mouse anti-human CD45 antibody (1:500; BD Biosciences, San José, CA, USA). L- and S-cone outer segments were detected using rabbit anti-red/green opsin (1:1200, AB5405; Chemicon-Millipore Iberica, Madrid, Spain) and goat anti-blue opsin (1:1000, N-20; OPN1SW; Santa Cruz Biotechnology, Heidelberg, Germany), respectively. Microglial cells were detected with a rabbit anti-Iba1 antibody (1:1000, 019-19741; Wako Chemicals, Neuss, Germany) and both astrocytes and Müller cells were detected by using antibodies against GFAP (1:250; goat anti-GFAP; C-19: sc-6170; Santa Cruz Biotechnology, Heidelberg Germany).

Secondary antibodies: donkey anti-goat Alexa Fluor^®^ 594 conjugate, donkey anti-mouse Alexa Fluor^®^ 594 conjugate and donkey anti-rabbit Alexa Fluor^®^ 488 conjugate were used. All the secondary antibodies were from Molecular Probes (Invitrogen Inc., Madrid, Spain) and diluted at 1:500.

### 4.6. Image Analysis

Retinal cross-sections were examined and photographed under a fluorescence microscope (Axioscop 2 Plus; Zeiss Mikroskopie, Jena, Germany) equipped with various filters and a digital high-resolution camera (ProgRes C10; Jenoptik, Jena, Germany). Eight images (530 × 390 µm) were taken per each retinal section, at four equidistant distances from the optic disk to the retinal periphery, four in the dorsal and four in the ventral retina. For this purpose, the length of the cross-sectioned hemiretina from the optic disk to the periphery was considered 100% and the pictures were taken at distances equal to 25%, 50%, 75% and 95% of the length. Images were further processed with Adobe^®^ Photoshop^®^ CS 6 (Adobe Systems, Inc., San Jose, CA, USA) when needed.

### 4.7. Quantification of Nuclei rows, Microglial Cells and GFAP Expression

In each animal, the number of nuclei rows of the outer nuclear layer (ONL), the number of microglial cells and the macroglial cell hypertrophy were quantified in three sagittal cross-sections containing the optic disk (from the nasal, central and temporal part of the optic disk). For each section eight photomicrographs (4 from the dorsal retina and 4 from the ventral retina, measuring 530 × 390 μm) situated at 25%, 50%, 75% and 95% of the total distance between the optic disk and the retinal periphery, were selected

The number of nuclei rows of the ONL were counted and averaged in the previously selected photographs in three regions (central, right and left) of each photomicrograph, thus obtaining a mean number of nuclei rows per picture, per retinal region analyzed and per animal. The number of microglial cells were counted in all retinal layers in each photomicrograph, these quantifications were pooled to obtain a mean number of microglial cells per retinal region and per animal. [24,74]. Macroglial cell hypertrophy was quantified using the tool “Histogram Analysis” of the software Image Pro Plus (IPP 5.1 for Windows; Media Cybernetics, Silver Spring, MD). This tool measured the intensity of GFAP fluorescence in each previously selected photograph, obtaining a plot and average of the intensity values along the photograph. To ensure measurement accuracy and to assure its uniformity, all the microphotographs were taken with the same gain and exposure time.

### 4.8. Statistics

Statistical analysis was carried out using GraphPad Prism^®^ for Windows (Version 5.01; GraphPad Software Inc., La Jolla, CA, EEUU). Variables were tested for normality. To compare more than two groups we used the analysis of variance (one-way ANOVA) followed by the Tukey post hoc test or the Kruskal–Wallis test. To compare two groups, we used the t-test or the Mann–Whitney test depending on the normality of the data. Differences were considered significant when *p* ≤ 0.05.

## 5. Conclusions

The injection of hBM-MSCs into the vitreous or in the subretinal space in immunosuppressed control or dystrophic rats does not cause local adverse effects such as tumorigenesis or immune rejection and the transplanted cells can survive for at least 2 weeks in the vitreous or in the subretinal space. Moreover, the IVI or SRI of hBM-MSCs to dystrophic P23H-1 and RCS rats did not elicit photoreceptor neuroprotective effects but caused a decrease in retinal gliosis, and thus it is our intention to continue investigating the possible therapeutic effects of these cells.

## Appendix A

**Table A1:** Number of nuclei rows of the ONL, microglial cells and quantification of GFAP expression in control and transplanted RCS and P23H-1 rats.

Nuclei Rows in ONL			Intravitreal Injection						Subretinal Injection					
Days	Control *n* = 6 (FTSS)		Right Eyes *n* = 12 (FTSS)		PBS *n* = 6 (FTSS)		hBM-MSCs *n* = 6 (FTSS)		Right Eyes *n* = 12 (FTSS)		PBS *n* = 6 (FTSS)		hBM-MSCs *n* = 6 (FTSS)	
	PVG	SD	RCS	P23H-1	RCS	P23H-1	RCS	P23H-1	RCS	P23H-1	RCS	P23H-1	RCS	P23H-1
28 (7)	10.4 ± 1.2	10.2 ± 1.1	6.1 ± 1.7 ^◊,†^	4 ± 1 ^◊,†^	6.7 ± 1.2^◊,†^	4.5 ± 1.3 ^◊,†^	5.9 ± 1.5 ^◊,†^	4.2 ± 0.8 ^◊,†^	6.3 ± 1.4 ^◊,†^	4.1 ± 0.7^◊,†^	6.2 ± 1.4 ^◊,†^	3.9 ± 1 ^◊,†^	5.7 ± 1.4 ^◊,†^	4.1 ± 0.9 ^◊,†^
36 (15)			5 ± 1 ^◊^	3.8 ± 0.7 ^◊^	5.2 ± 1.4^◊^	3.5 ± 0.9 ^◊^	5.1 ± 1.7 ^◊^	3.7 ± 0.8 ^◊^	5.5 ± 1 ^◊^	3.8 ± 0.5 ^◊^	5 ± 1.7 ^◊^	3.1 ± 0.8 ^◊^	5 ± 2.1 ^◊^	3.7 ± 1.2 ^◊^
51 (30)			3.8 ± 0.9 ^○^	3.5 ± 0.5 ^○^	4.6 ± 1.5^○,◊^	3.2 ± 0.4 ^○,◊^	4.2 ± 1.3 ^○,◊^	3.4 ± 0.6 ^○,◊^	3.4 ± 1.2 ^○,□,◊^	3.1 ± 0.8 ^○,◊^	3.7 ± 1.1 ^○,◊^	2.7 ± 0.5 ^○,◊^	4.6 ± 1.4 ^○,◊^	3.2 ± 0.9 ^○,◊^
81 (60)	10.1 ± 1	10.4 ± 1.2	1.2 ± 0.3 ^○,◊,†^	3.1 ± 0.8 ^○,◊,†^	1.5 ± 0.5○□^◊,†^	2.8 ± 0.6 ^○,◊,†^	1.6 ± 0.4 ^○,□,◊,†^	3 ± 0.7 ^○,◊,†^	1.3 ± 0.5 ^○,□,◊,†^	2.9 ± 0.6 ^○,◊,†^	1.4 ± 0.3 ^○,□,◊,†^	2 ± 0.7 ^○,◊,†^	1.5 ± 0.2 ^○,□,◊,†^	3 ± 0.8 ^○,◊,†^
**Iba-1^+^ cells**														
28 (7)	11.3 ± 6.2	10.6 ± 4	22.5 ± 3.9 ^†^	18 ± 3.5 ^†^	23.5 ± 4.1 ^†^	17 ± 4.5 ^†^	23.4 ± 5.6 ^†^	19 ± 5 ^†^	24.1 ± 5 ^†^	22.3 ± 3.9 ^†^	22.8 ± 5 ^†^	19 ± 3.9 ^†^	22 ± 6.2 ^†^	23 ± 3.6 ^†^
36 (15)			26.4 ± 4.3	20.9 ± 4.4	24.5 ± 5.1	21.2 ± 4.1	23.8 ± 3.5	22.1 ± 4.9	26.3 ± 6.1	25.3 ± 5.1	23.9 ± 4.9	22.9 ± 4.9	23.5 ± 3.8	24.3 ± 4.7
51 (30)			25.1 ± 5.1	21.75 ± 3.5	26.4 ± 6.2	23.6 ± 4.6	25 ± 4.9	23 ± 3.1	24.4 ± 4.8	24.4 ± 4.7	25.9 ± 5.3	24.3 ± 4.2	23.2 ± 5.1	25.5 ± 7.9
81 (60)	13.7 ± 4.7	14 ± 4.7	24.9 ± 6 ^†^	22.3 ± 5.3 ^†^	27.1 ± 5.8 ^†^	23.9 ± 5.1 ^†^	24.4 ± 4.2 ^†^	23.3 ± 4.1 ^†^	21 ± 5 ^†^	25.2 ± 5.6 ^†^	26.7 ± 5.7 ^†^	24.9 ± 4.9 ^†^	22 ± 4.7 ^†^	24 ± 6.1 ^†^
**RFU (GFAP expression)**														
28 (7)	1567 ± 8.7	1736 ± 96	3856 ± 90 ^†^	3987 ± 99 ^†^	3767 ± 102 ^◊,†^	3905 ± 88 ^◊,†^	3025 ± 120 ^◊,^*^,†,€^	2644 ± 125 ^◊,^*^,†,€^	3861 ± 88 ^†^	3799 ± 98 ^†^	3955 ± 95 ^†^	3832 ± 73 ^†^	2825 ± 93^◊^*^†€^	2444 ± 80 ^◊,^*^,†,€^
36 (15)			8741 ± 85 ^○,◊^	3738 ± 85 ^◊^	8978 ± 112 ^○,◊^	3882 ± 102 ^◊^	6273 ± 145 ^○,^*^,□,◊,€^	2788 ± 87 *^,□,◊,€^	8758 ± 121 ^○,◊^	3679 ± 89 ^◊^	8797 ± 103 ^○,◊^	3922 ± 88^◊^	5880 ± 113 ^○,^*^,□,◊,€^	2888 ± 120 ^○,^*^,□,◊,€^
51 (30)			9900 ± 180 ^○,◊^	3934 ± 80 ^◊^	9855 ± 159 ^○,◊^	4110 ± 109 ^○,◊^	6549 ± 136 ^○,^*^,□,◊,€^	3130 ± 91 ^○,^*^,□,◊^	9906 ± 183 ^○,◊^	3958 ± 106 ^◊^	9979 ± 172 ^○,◊^	4096 ± 97 ^○,◊^	6251 ± 87 ^○,^*^,□,◊,€^	3230 ± 99 ^○,^*^,□,◊^
81 (60)	1987 ± 85	1856 ± 85	9889 ± 195 ^○,◊,†^	3893 ± 95 ^◊,†^	9946 ± 185 ^○,◊,†^	4001 ± 125 ^○,◊,†^	6834 ± 119 *^,□,◊,†,€^	3250 ± 101 ^○,^*^,□,◊,†,€^	9872 ± 174 ^○,◊,†^	3798 ± 90 ^◊,†^	1055 ± 169 ^○,◊,†^	3989 ± 115 ^○,◊,†^	6365 ± 111 ^○,^*^,◊,†,€^	3550 ± 121 ^○,^*^,□,◊,†,€^

Days column: First number (without parenthesis) corresponds to animal age (post-natal day) and number in parenthesis corresponds to days after injection. FTSS = For each Time point Studied and Strain. *n* = number of eyes. ^○^ Significant differences with baseline value (28 days) *p* < 0.005 U Mann Whitney or *t*-test. * Significant difference with the right eyes of the same strain at the same age. U Mann Whitney or *t*-test *p* < 0.001. ^□^ Significant difference with previous survival interval. One way ANOVA or Kruskal Wallis *p* < 0.0001. ^◊^ Significant difference between Pigmented (PVG or RCS) and Albino (SD or P23H-1) rats at the same age and injection protocol (PVG were compared to SD and RCS to P23H-1). U Mann Whitney or *t*-test *p* < 0.001. ^†^ Significant difference with the respective control eyes at the same age. U Mann Whitney or t-test *p* < 0.001. ^€^ Significant differences between intreavitreal and subretinal injection at the same age in the same strain. *p* < 0.005 U Mann Whitney or *t*-test.
